# The acute respiratory response to blood‐flow restriction resistance exercise in healthy adults: A randomized crossover trial

**DOI:** 10.14814/phy2.70968

**Published:** 2026-06-12

**Authors:** Manuel Kuhn, Dario Kohlbrenner, Adrian Kläy, Malcolm Kohler, Alea Gheza, Laura C. Mayer, Thomas Radtke, Sarah R. Haile, Diego M. Baur, Noriane A. Sievi, Christian F. Clarenbach

**Affiliations:** ^1^ Faculty of Medicine University of Zurich Zurich Switzerland; ^2^ Department of Pulmonology University Hospital Zurich Zurich Switzerland; ^3^ Epidemiology, Biostatistics and Prevention Institute University of Zurich Zurich Switzerland

**Keywords:** blood flow restriction, healthy individuals, respiratory response, strength exercise

## Abstract

This study compared acute respiratory responses to low‐load blood flow restriction resistance exercise (LL‐BFR) and traditional high‐load resistance exercise (HL‐TRA) in healthy individuals. A randomized crossover design with a 48‐h washout was used. Participants completed LL‐BFR (30% one repetition maximum [1RM], 70% limb occlusion pressure [LOP]) and HL‐TRA (70% 1RM, no occlusion) on a leg press. HL‐TRA involved 3 sets of 12 repetitions; LL‐BFR included 4 sets (15, 30). Respiratory parameters were measured breath‐by‐breath. Ratings of perceived breathing effort and leg exertion (0–10 scale) were recorded. Data were analyzed using linear mixed models. Twenty‐four participants (8 males, 16 females) completed the study. LL‐BFR resulted in lower minute ventilation (VE, primary outcome) by −3.32 L/min (95% CI: −4.55 to −2.08), oxygen consumption by −0.12 L/min (95% CI: −0.15 to −0.09), carbon dioxide production by −0.16 L/min (95% CI: −0.19 to −0.13), and perceived breathing effort by −1.2 units (95% CI: −1.5 to −0.8) compared to HL‐TRA. Perceived leg exertion was higher with LL‐BFR (1.8 units; 95% CI: 1.3 to 2.2). LL‐BFR elicited lower respiratory responses and reduced perceived breathing effort than HL‐TRA, despite greater leg discomfort.

## Graphical abstract

LL‐BFR resistance exercise offers a promising alternative to traditional high‐load resistance training by reducing respiratory demand and perceived breathing effort, making it particularly beneficial for individuals with compromised respiratory function.

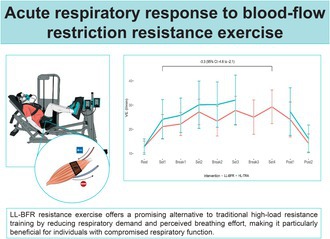

## 1 INTRODUCTION

Resistance training is fundamental for enhancing muscle strength and overall physical function. Current guidelines recommend the use of high training loads, typically greater than 65% of one‐repetition maximum (1RM), to induce optimal strength adaptations (Ratamess et al., 2009). However, these intensities are often not feasible for vulnerable populations, including individuals with frailty, limited resistance training experience, or chronic conditions such as cardiopulmonary disease. In these populations, high‐load resistance exercise may provoke excessive exertion, heightened symptom burden, or safety concerns, leading to the use of low‐load training protocols that are frequently insufficient to induce meaningful strength gains. Consequently, alternative training strategies that enable effective strength adaptations at lower external loads are of considerable clinical and rehabilitative interest.

Low‐load resistance exercise combined with blood flow restriction (LL‐BFR) has emerged as a promising alternative to traditional high‐load resistance training (HL‐TRA). LL‐BFR involves the application of an inflatable cuff around the proximal portion of the exercising limb to partially restrict arterial inflow and venous outflow, thereby creating a hypoxic and metabolically stressful intramuscular environment (Hjortshoej et al., 2023; Labata‐Lezaun et al., 2022; Loenneke et al., 2014; Scott et al., 2015). This approach has been shown to induce substantial improvements in muscle strength and hypertrophy using loads as low as 20%–30% 1RM, with adaptations comparable to those achieved through HL‐TRA (Chang et al., 2023; Gronfeldt et al., 2020; Labata‐Lezaun et al., 2022; Patterson, Hughes, Warmington, Burr, et al., 2019). As a result, LL‐BFR is increasingly considered a viable training modality for individuals who are unable to tolerate external high loads.

While the muscular adaptations to LL‐BFR are well documented, its acute respiratory demands defined by ventilatory and metabolic responses during resistance exercise remain insufficiently characterized. Previous studies have investigated cardiovascular and hemodynamic responses to blood flow restriction exercise, including heart rate and blood pressure responses, as well as perceptual strain (de Queiros et al., 2022; Lemos et al., 2022; Parkington et al., 2022; Rossow et al., 2012). However, fewer studies have examined respiratory parameters such as minute ventilation (V̇E), oxygen uptake (V̇O_2_), and carbon dioxide production (V̇CO_2_) during LL‐BFR, particularly in comparison with HL‐TRA. Characterizing these responses in healthy individuals under conditions that reflect typical clinical practice allows a clearer description of the respiratory demands associated with LL‐BFR. Respiratory demand contributes to perceived exertion and exercise tolerance and, while unlikely to limit performance in healthy individuals, may become relevant in populations with impaired pulmonary function. Therefore, characterizing the acute respiratory responses to LL‐BFR represents an important step toward its safe and informed application in rehabilitation settings.

The present study was designed to compare the acute respiratory and perceptual responses to LL‐BFR and conventional HL‐TRA under conditions that reflect how these approaches are typically applied. By examining these responses in healthy adults, we aimed to provide a controlled comparison of the overall physiological demands associated with each training modality. Accordingly, this randomized crossover study compared the acute respiratory and perceptual responses to LL‐BFR resistance exercise and conventional HL‐TRA in healthy adults. The primary outcome was minute ventilation (V̇E), with secondary outcomes including V̇O_2_, V̇CO_2_, heart rate, and ratings of perceived exertion. We hypothesized that LL‐BFR would elicit lower respiratory demands, defined by ventilatory and metabolic responses than those observed during HL‐TRA, reflecting the decreased external load associated with BFR. By directly comparing these two resistance exercise modalities, this study aims to advance understanding of the acute physiological demands of LL‐BFR and support its potential role as a safe and effective alternative to high‐load resistance training.

## 2 MATERIALS AND METHODS

### 2.1 Individuals

Participants were recruited between September 2022 and June 2023. Eligibility criteria required participants to be at least 18 years old and healthy. Exclusion criteria included individuals experiencing pain during exercise of any etiology, a history of thromboembolic events in the lower extremities, or a resting systolic blood pressure (BP) below 100 mmHg. Additionally, pregnant individuals and those with a mental or physical disability that impeded their ability to provide informed consent or comply with the study protocol were excluded. The study was conducted in accordance with the declaration of Helsinki and all participants provided written informed consent. The Ethics Committee of the Canton of Zurich approved the study (EK‐ZH‐NR: 2021‐02038). The study was registered on clinicaltrials.gov (NCT05163600).

### 2.2 Experimental design

The study was designed as a monocentric randomized crossover study (AB / BA). Minute ventilation (V̇E) was selected as the primary outcome because it represents the overall ventilatory response to exercise and is a key determinant of respiratory effort and perceived exertion. While V̇E is particularly important in populations with impaired pulmonary function, such as individuals with chronic lung disease, it also provides a sensitive measure of acute respiratory demand in healthy adults. By quantifying V̇E during LL‐BFR and HL‐TRA, we can directly assess the respiratory burden imposed by each resistance exercise modality. This choice aligns with the study's translational goals, as understanding ventilatory demands in healthy participants provides a foundation for predicting tolerability and safety in clinical populations with limited ventilatory reserve. Single exercise bouts induce transient perturbations in physiological homeostasis. However, they do not elicit sustained adaptations in human physiology, thereby rendering them suitable for crossover trials. Secondary outcomes included oxygen uptake (VO_2_), heart rate (HR), carbon dioxide output (VCO_2_), tidal volume (VT), and rating of perceived exertion (RPE) for the legs and breathlessness.

Three hospital visits were included in the study, each lasting approximately 1 h. During Visit 1, participants underwent screening to confirm eligibility and provided written informed consent. Height, weight, and peak cardiopulmonary values were assessed using a cardiopulmonary exercise test (CPET). Randomization into one of two sequences (LL‐BFR/HL‐TRA or HL‐TRA/LL‐BFR) was performed using block randomization with block sizes of 2–4, ensuring balanced group allocation. The study setting was individually adapted to ensure that equipment and face masks fit properly (e.g., saddle height, handlebar reach). Visits 2 and 3 comprised the exercise sessions. Participants assigned to the HL‐TRA/LL‐BFR sequence performed high‐load resistance training (HL‐TRA) without blood flow restriction (BFR) during Visit 2, followed by low‐load resistance training with continuous BFR (LL‐BFR) on Visit 3. Conversely, those in the LL‐BFR/HL‐TRA sequence completed the conditions in reverse order. To determine appropriate exercise intensities, a one‐repetition maximum (1RM) test was conducted before the exercises in Visit 2. All measurements were conducted at the same time of day for each participant to minimize circadian variability.

**FIGURE 1:**
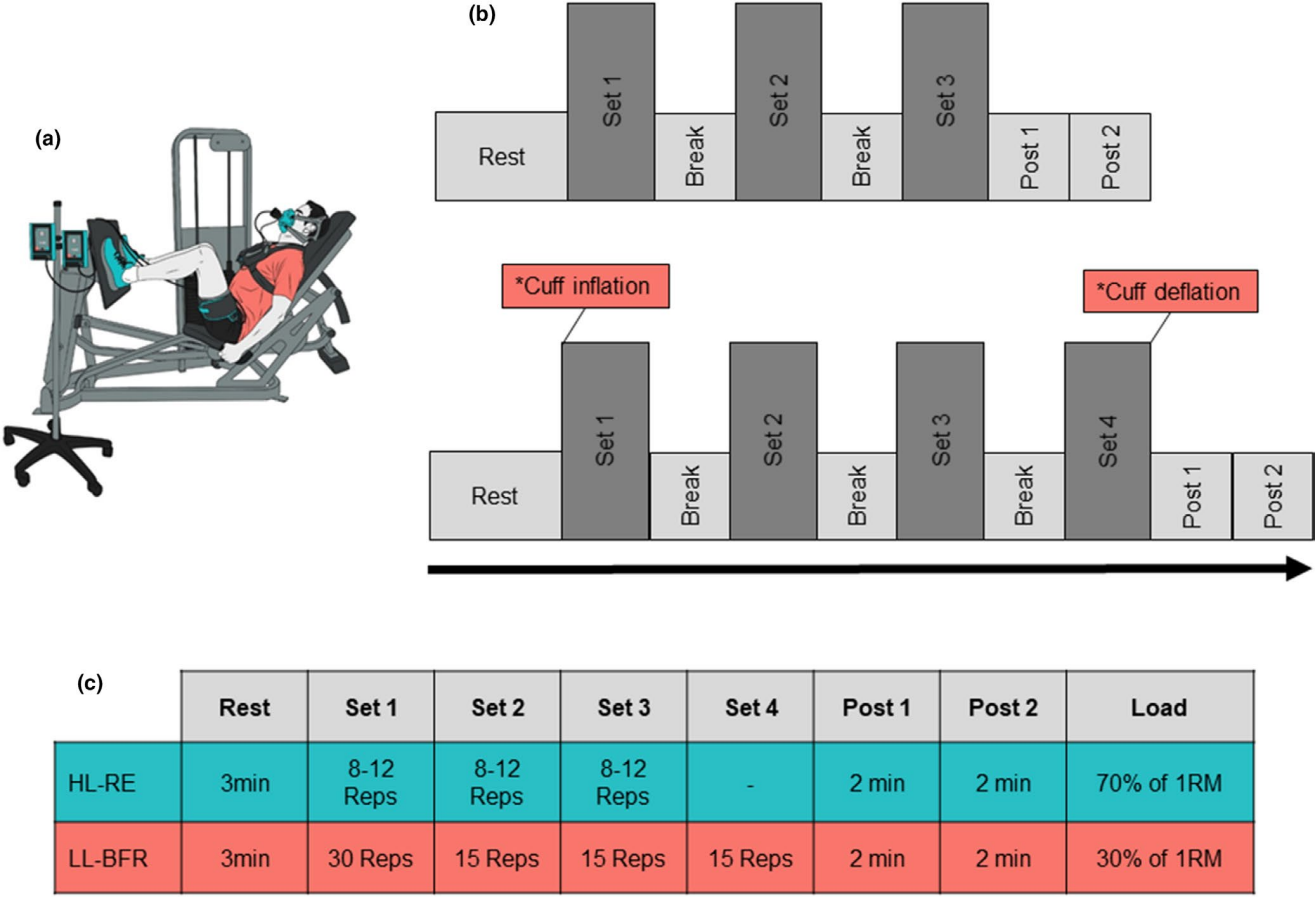
Overview of the resistance exercise protocols. (a) Schematic of the study setup showing the application of BFR cuffs, the use of a leg press machine, and breath‐by‐breath gas analysis equipment. (b) Timeline detailing the sequence of sets, breaks, and post‐exercise periods for high‐load resistance exercise (HL‐TRA) and low‐load blood flow restricted exercise (LL‐BFR). (c) Comprehensive summary of load and repetition parameters for both LL‐BFR and HL‐TRA protocols. 1RM, one repetition maximum; Reps, repetitions; LL‐BFR, low‐load blood flow restricted exercise; HL‐TRA, high‐load resistance exercise. *Denotes cuff inflation/deflation, specific to LL‐BFR.

The resistance exercise load was either high or low, but the exercise volume during each visit was relatively low in both cases. Therefore, it is assumed that full recovery would be achieved within 48 h. (Tan, 1999) To standardize conditions, participants refrained from strenuous physical activity for 48 h before each visit and maintained consistent daily routines regarding diet, hydration, and sleep. A graphical representation of the study design is provided in Figure 1.

### 2.3 Cardiopulmonary exercise testing (visit 1)

Upon arrival at the hospital, the exact test protocol was explained to the participant. Volume and gas calibrations were performed before each test according to the manufacturer. CPET was performed in accordance with published guidelines (Medicine ACoS, 2010). Respiratory parameters (VE, VO_2_, carbon dioxide output [VCO_2_], tidal volume [VT], breathing rate [BR]) were collected breath‐by‐breath using a metabolic cart (Metamax 3b, Cortex Biophysik GmbH, Leipzig, Germany). In addition, continuous peripheral oxygen saturation (SpO_2_) with pulse oximetry (Wrist Ox2 3150, Nonin Medical, Minnesota, USA) and heart rate (HR in beats per min [bpm]) with a chest belt (H10, Polar Electro OY, Kempele, Finnland) were recorded during CPET. Before and after the CPET, blood pressure (BP) and ratings of perceived breathlessness and leg exertion (RPE, numeric rating scale ranging from 0 to 10 with 0 representing “no leg fatigue” and “no shortness of breath”, and 10 representing “maximum leg fatigue” and “maximum shortness of breath”, respectively) (Borg et al., 2010) were measured.

Initially, individuals rested for 3 min on the cycle ergometer (ergoselect 200, ergoline GmbH, Bitz, Germany) for collection of resting respiratory gas exchange and HR data, followed by a 3 min warm‐up period of unloaded pedaling at 60 revolutions per minute (rpm). Subsequently, an incremental ramp exercise test was performed until exhaustion. The baseline load (50–100 W) and the increments in load per min (20 to 30 W) were individualized, depending on the self‐reported training status of each individual and aiming for a test duration (i.e., the incremental ramp phase) of 8 to 12 min. The last 15 s of the incremental ramp exercise was used to determine VO_2peak_.

### 2.4 One‐repetition maximum testing (visit 2)

Before the primary exercise session during the second visit, each participant completed a one‐repetition maximum (1RM) test to determine the exercise load for the LL‐BFR and HL‐TRA conditions. Participants began with a warm‐up on the leg press machine (Leg Press VR2 and Eagle, Cybex International, Medway, Massachusetts, USA), performing 15 to 20 repetitions with 30% of the estimated 1RM. After the warm‐up, the load was adjusted based on the participant's self‐perceived capacity, with increases ranging from 1 to 20 kg (Grgic et al., 2020). The 1RM was assessed on the leg press using the repetition‐to‐failure method. Participants were instructed to lift a predetermined load until failure. If the number of repetitions completed fell within six, the 1RM was estimated. If more than six repetitions were achieved, the load was increased for subsequent attempts (Dohoney et al., 2002; Liguori et al., 2021). The rest period between attempts was 1 min (Levinger et al., 2009). The rest period between 1 RM testing and main resistance exercise was 15 min (Hernández‐Lougedo et al., 2022; Hu et al., 2025).

### 2.5 Main resistance exercise trial (visit 2 and 3)

Participants performed a warm‐up consisting of 15–20 repetitions at 30% of their estimated 1RM. Following the warm‐up, all necessary assessment equipment and cables were attached to the participants, a process that took approximately 10 min. Prior to initiating the exercise protocol, participants rested on the leg press machine for 2 min to obtain baseline measurements. Following this period, they commenced the exercise regimen. The HL‐TRA protocol was designed in accordance with established resistance training guidelines and consisted of 3 sets of 8–12 repetitions performed at 70% of 1RM using a 1–0–1‐0 tempo (1 s concentric phase, no pause, 1 s eccentric phase, no pause) (Liguori et al., 2021). The inter‐set rest interval was 1 min and 30 s (i.e., Break 1 and Break 2).

The LL‐BFR protocol followed a widely used and empirically supported scheme (Patterson, Hughes, Warmington, et al., 2019). Participants performed 30 repetitions at 30% 1RM in the first set, followed by three sets of 15 repetitions each, with one‐minute inter‐set rest (i.e., Break 1, Break 2, and Break 3). Limb occlusion pressure (LOP) was set to 70%. The BFR cuffs were applied to the most proximal part of both legs and were inflated at the start of the first set after the baseline resting, remaining inflated until the end of the fourth set. For an overview of the resistance protocols, see Figure 1.

### 2.6 Measurements during resistance exercise

During exercise, ventilatory gas exchange variables were measured breath‐by‐breath using a metabolic chart. In addition, continuous saturation of peripheral oxygen (SpO_2_) with pulse oximetry (Nonin Wrist Ox2 3150, Somnovum, Oberentfelden, Switzerland) and HR data with a chest belt (Polar Electro OY, Kempele, Finnland) were recorded during the whole exercise protocol. RPE Leg and Breathing was measured immediately after each set and 2 and 4 min after the last set (i.e., Post1 and Post2).

### 2.7 Limb occlusion pressure (LOP)

The limb occlusion pressure (LOP) was individually determined using an automatic tourniquet system (PTS for BFR, Delfi Medical Innovations Inc., Vancouver, Canada) while participants were in a relaxed supine position. Inflatable cuffs (Easy Fit BFR 11.5 × 86 cm, Delfi Medical Innovations Inc., Vancouver, Canada) were positioned around the most proximal part of each thigh, and the LOP was measured separately for each limb. During LL‐BFR, the system automatically applied cuff pressures equivalent to 70% of the LOP, with continuous adaptation to each limb separately throughout the four sets and three breaks. At the end of Set4, the cuffs were deflated (Hughes et al., 2018).

### 2.8 Statistical analysis

The data are presented as mean (standard deviation, SD), unless otherwise noted. Linear mixed‐effects models were used to compare respiratory parameters, including the primary outcome V̇E, as well as secondary outcomes V̇O_2_, V̇CO_2_, VT, BR, SpO_2_, HR, and RPE, between HL‐TRA and LL‐BFR across exercise phases.

Two complementary linear mixed‐effects models were fitted for each outcome. First, a model including condition as the sole fixed effect was used to estimate the overall between‐condition differences averaged across exercise phases, excluding rest and recovery periods. These results are presented in Table 2. Second, a condition × time interaction model (outcome ~ condition × time + (1|participant)) was used to evaluate whether between‐condition differences varied across exercise phases. These phase‐specific analyses are presented in Table 3 and were based on respiratory parameters, SpO_2_, RPE, and HR data averaged over the final 20 s of each respective phase to account for the dynamic nature of respiratory responses during resistance exercise, where a physiological steady state may not be achieved.

The models included fixed effects for condition, time, and the condition × time interaction, adjusted for treatment sequence and period, with a random intercept for each participant to account for the repeated‐measures crossover design. Because HL‐TRA consisted of 3 sets and 2 breaks, whereas LL‐BFR consisted of 4 sets and 3 breaks, the dataset represented an inherently unbalanced repeated‐measures structure. Linear mixed‐effects modeling accommodates this directly, as phases absent in a given condition (e.g., Set4 and Break3 during HL‐TRA) are treated as structurally missing and are not imputed or excluded from the likelihood estimation. Time was treated as a categorical fixed effect, with each exercise phase (“Set1”, “Set2”, “Set3”, “Set4”, “Break1”, “Break2”, “Break3”, “Post1” and “Post2”) modeled as a separate level. Restricted maximum likelihood estimation (REML) was used to fit all mixed‐effects models.

The study was powered to detect a moderate effect size (0.6) in the primary endpoint V̇E between the two exercise regimens. Assuming 80% power and a two‐sided significance level of 5%, a sample size of 24 participants was required. After Visit 1, eligible participants were randomly assigned to their intervention sequence using computer‐generated permuted block randomization with random block sizes of 2 to 4 implemented with the blockrand package in R (Snow, 2020).

The statistical analyses were performed using R‐4.3.1 on Windows (R Core Team, 2023, R Foundation for Statistical Computing, Vienna, Austria).

## 3 RESULTS

### 3.1 Sample characteristics

**FIGURE 2:**
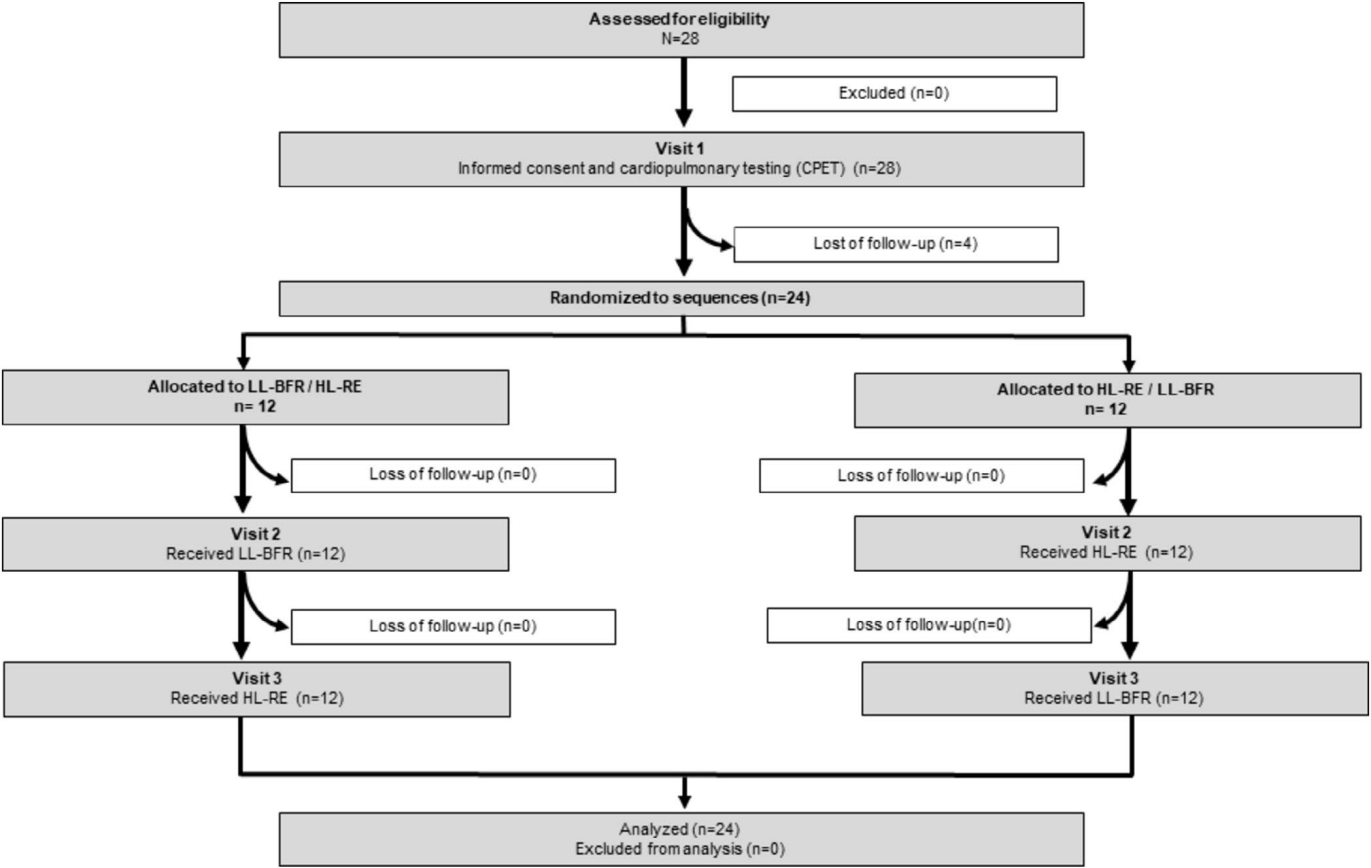
Flow chart. LL‐BFR: low‐load blood flow restricted exercise; HL‐TRA: high‐load resistance exercise.

Twenty‐four individuals (16 females, 8 males) were included in the study. All 24 individuals completed all examinations and were included in the final analysis (Figure 2). Characteristics of the study population, stratified by test sequence, are given in Table 1. During the HL‐TRA condition, individuals performed an average of 35.8 repetitions (±1), with a total exercise volume of 3769 kg (±1244). In contrast, under the LL‐BFR condition, individuals completed an average of 74.6 repetitions (±2), with a total exercise volume of 3378 kg (±1152).

**TABLE 1:** Participant characteristics.

	LL‐BFR/HL‐TRA	HL‐TRA/LL‐BFR	Overall
	(*n* = 12)	(*n* = 12)	(*n* = 24)
Sex			
Male	4 (33.3%)	4 (33.3%)	8 (33.3%)
Female	8 (66.7%)	8 (66.7%)	16 (66.7%)
Age (years)	34.0 [26.3, 35.5]	29.5 [21.8, 31.5]	30.5 [24.0, 34.5]
BMI (kg/m^2^)	23.1 [20.8, 24.4]	22.5 [21.2, 25.1]	22.8 [20.9, 24.9]
LOP (mmHg)			
Left leg	170 [162, 181]	188 [159, 204]	174 [160, 199]
Right leg	171 [157, 189]	176 [158, 210]	174 [158, 195]
1 repetition max (kg)	132 [113, 145]	144 [129, 174]	134 [124, 166]
VE_peak_ (L/min)	113 [90.2, 130]	117 [100, 142]	117 [95.7, 133]
VO_2peak_ (mL/min/kg)	46 [40.4, 49.0]	48.5 [39.8, 52.3]	47.5 [40.0, 52.0]
VO_2peak_ (L/min)	3.23 [2.48, 3.66]	3.14 [2.76, 3.64]	3.23 [2.65, 3.66]
VCO_2peak_ (L/min)	3.49 [2.76, 4.11]	3.37 [3.02, 4.09]	3.40 [2.88, 4.09]
Heart rate_peak_ (bpm)	180 [175, 190]	180 [173, 185]	188 [173, 188]

*Note*: All data are presented as median [25th and 75th interquartile range] or *n* (%).

Abbreviations: BMI, Body mass index; LOP, Limb occlusion pressure; VCO_2peak_, peak carbon dioxide output; VE_peak_, Peak ventilatory equivalent (respiratory minute volume); VO_2_peak, Peak oxygen consumption.

### 3.2 Acute respiratory and perceptual response

Compared to HL‐TRA, the linear mixed model showed consistently lower values across several key respiratory parameters in LL‐BFR. Specifically, the mean differences observed for LL‐BFR relative to HL‐TRA included lower VE by −3.32 L/min (95% CI: −4.55 to −2.08), VO_2_ by −0.12 L/min (95% CI: −0.15 to −0.09), VCO_2_ by −0.16 L/min (95% CI: −0.20 to −0.13), VT by −0.14 L (95% CI: −0.20 to −0.09), HR by −4.4 bpm (95% CI: −6.8 to −2.0), and RPE breathing by −1.2 points (95% CI: −1.50 to −0.80), as detailed in Table 2. In contrast, mean differences between HL‐TRA and LL‐BFR indicated an increase in RPE Leg by 1.76 points (95% CI: 1.32 to 2.20) and a modest rise in BR by 0.4 breaths/min (95% CI: −0.50 to 1.20) for LL‐BFR. Significant condition × time interactions were observed for several respiratory and perceptual variables, indicating that the magnitude of differences between HL‐TRA and LL‐BFR varied across exercise phases.

**TABLE 2:** Linear mixed model analysis of mean differences in ventilation (VE) and secondary outcomes between high‐load resistance exercise (HL‐TRA) and low‐load blood flow restriction exercise (LL‐BFR) over Set1, Set2, Set3, Set4, Break1, Break2, and Break3.

	HL‐TRA (*n* = 24)	LL‐BFR (*n* = 24)	Mean differences (*n* = 24)	% differences	*p*‐value
Primary end point					
VE L/min	28.5 (25.9 to 31.1)	25.1 (22.5–27.7)	−3.32 (−4.55 to −2.08)	−11.7	<0.001
Secondary end points					
VO_2_ mL/min/kg	13.1 (12.1–14.1)	11.4 (10.3–12.4)	−1.74 (−2.20 to −1.28)	−13.3	<0.001
VO_2_ L/min	0.9 (0.8–1.0)	0.78 (0.7–0.9)	−0.12 (−0.15 to −0.09)	−13.3	<0.001
VCO_2_ L/min	0.9 (0.8–1.0)	0.7 (0.6–0.8)	−0.16 (−0.20 to −0.13)	−18.4	<0.001
RER	1.0 (1.0–1.0)	0.9 (0.9–0.9)	−0.09 (−0.11 to −0.07)	−9.0	<0.001
VE/VCO_2_	26.9 (23.9–29.9)	29.4 (26.5–32.4)	2.52 (1.54 to 3.51)	9.4	<0.001
VT L/min	1.3 (1.1–1.4)	1.1 (1.0–1.3)	−0.14 (−0.20 to −0.09)	−11	<0.001
BR (breaths per min)	23.7 (21.7–25.7)	24.1 (22.1–26.1)	0.40 (−0.50 to 1.20)	1.7	0.360
Heart rate (bpm)	104 (100–108)	100 (96–104)	−4.40 (−6.80 to −2.00)	−4.2	<0.001
SpO_2_ (%)	96.5 (96.1–97.0)	96.8 (96.3–97.2)	0.20 (0.00 to 0.40)	0.2	0.014
RPE leg (0–10)	5.1 (4.4–5.8)	6.9 (6.2–7.5)	1.76 (1.32 to 2.20)	34.5	<0.001
RPE breathing (0–10)	3.7 (3.1–4.2)	2.5 (2.0–3.0)	−1.20 (−1.50 to −0.80)	−32.4	0.011

*Note*: Data are presented as means with corresponding 95% confidence intervals, mean differences with corresponding 95% confidence intervals, and percent differences with corresponding 95% confidence intervals. Positive coefficients indicate that high‐load resistance exercise resulted in larger measurements compared to low‐load blood flow restriction exercise.

Abbreviations: BR, breathing rate; RPE Breathing, ratings of perceived breathing on a scale of 0–10 (0 no shortness of breath 10 maximum shortness of breath); RPE Leg, rating of perceived leg exertion on a scale of 0–10 (0 no fatigue; 10 maximum fatigue); SpO_2_, peripheral oxygen saturation; VCO_2_, carbon dioxide output; VE, ventilation; VO_2_, oxygen consumption; VT, tidal volume.

**FIGURE 3:**
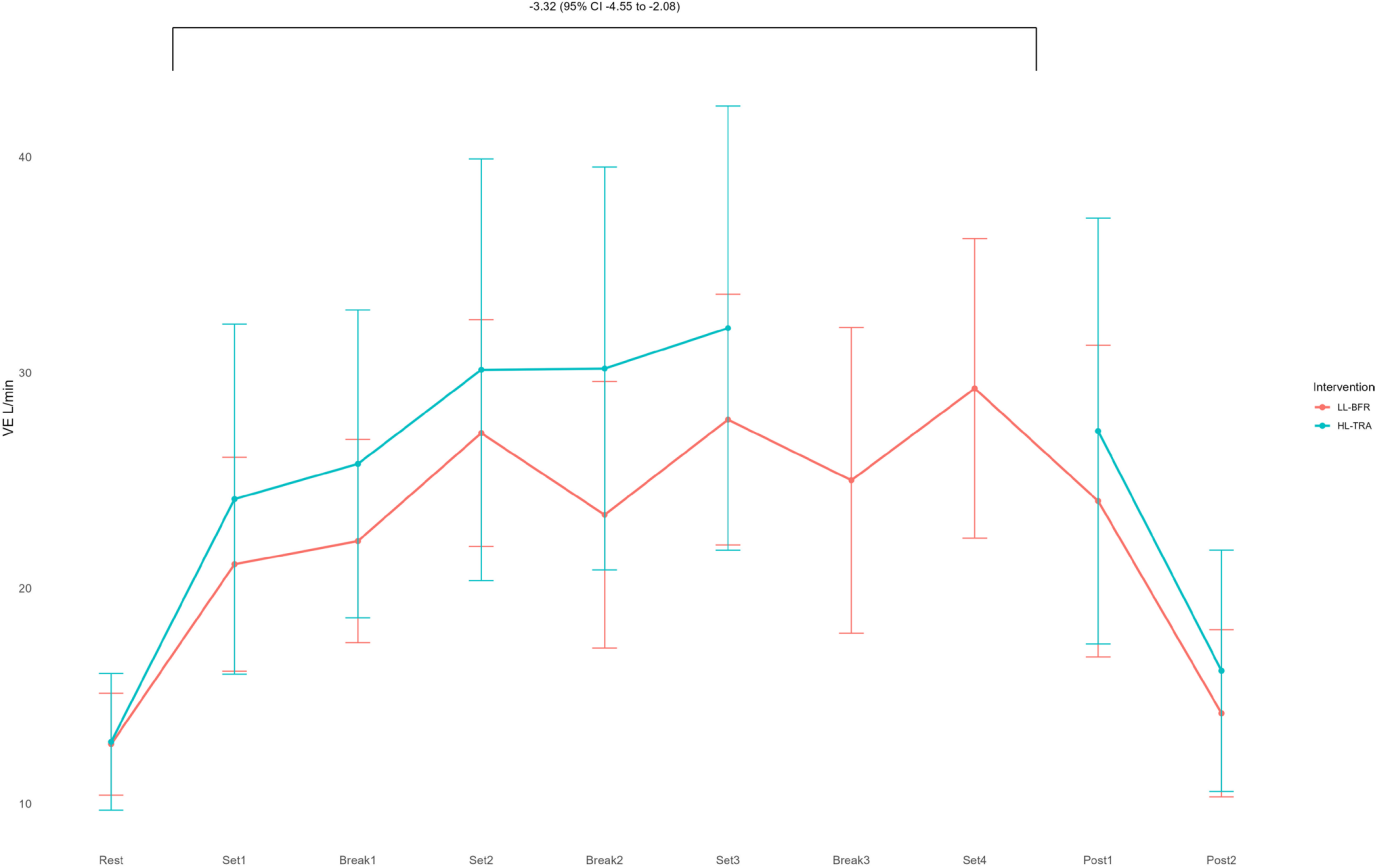
Acute cardiorespiratory responses during resistance exercise with low‐load blood flow restriction (LL‐BFR) and high‐load traditional resistance exercise (HL‐TRA). Panel shows ventilation (VE). Data are presented as mean ± standard deviation for each condition: LL‐BFR (red line) and HL‐TRA (blue line). Brackets with mean differences and 95% confidence intervals (CI) indicate statistically significant between‐condition differences across the exercise period.

**FIGURE 4:**
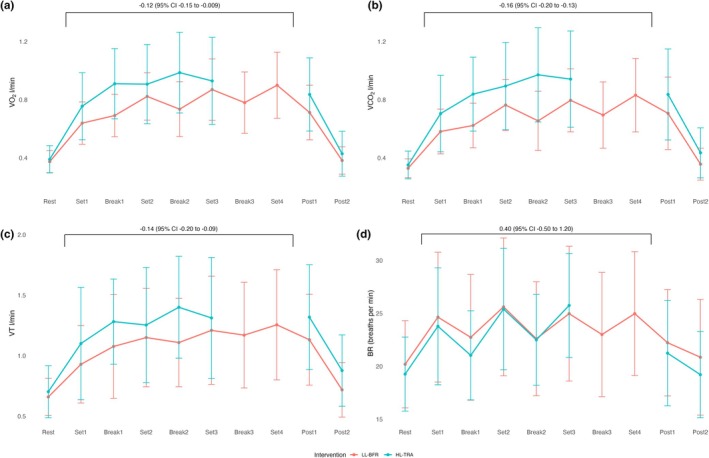
Acute cardiorespiratory responses during resistance exercise with low‐load blood flow restriction (LL‐BFR) and high‐load traditional resistance exercise (HL‐TRA). Panel A shows oxygen uptake (VO_2_), Panel B shows carbon dioxide output (VCO_2_), Panel C displays tidal volume (VT), and Panel D represents breathing rate (BR) across time points. Measurements were taken at rest, during each exercise set (Set1–Set4), during intra‐session breaks (Break1–Break3), and during post‐exercise recovery (Post1–Post2). Data are presented as mean ± standard deviation for each condition: LL‐BFR (red line) and HL‐TRA (blue line). Brackets with mean differences and 95% confidence intervals (CI) indicate statistically significant between‐condition differences across the exercise period.

**FIGURE 5:**
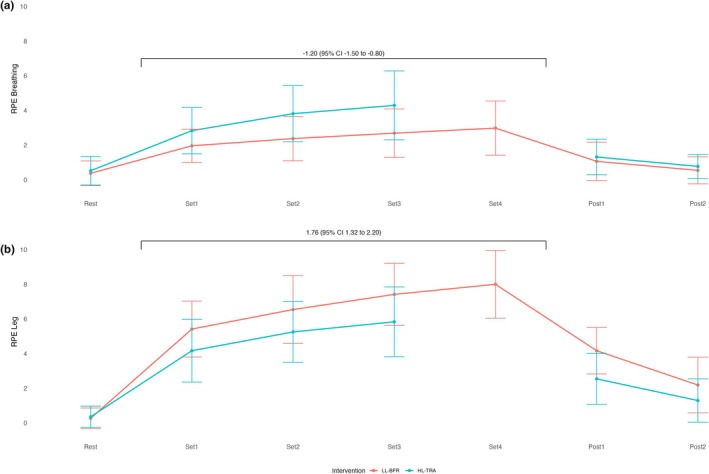
Perceived exertion during low‐load blood flow restriction (LL‐BFR) and high‐load traditional resistance exercise (HL‐TRA). Panel A shows the rating of perceived exertion for breathing (RPE Breathing), and Panel B shows the rating of perceived exertion for the legs (RPE Leg) assessed at rest, during each exercise set (Set1–Set4), and post‐exercise recovery (Post1 and Post2). Data are presented as mean ± standard deviation for each condition: LL‐BFR (red line) and HL‐TRA (blue line). Brackets with mean differences and 95% confidence intervals (CI) indicate statistically significant between‐condition differences across the exercise period.

Figures 3, 4, 5 provide a detailed comparison of the respiratory responses between HL‐TRA and LL‐BFR across various exercise phases, including sets, breaks, rest, and post‐exercise periods. To explore the underlying causes of these observed differences, post hoc pairwise comparisons were conducted for each exercise phase, as outlined in Table 3. To summarize, in the LL‐BFR condition, VE, VO_2_, and VCO_2_ were consistently lower across all sets and breaks, with the largest reductions during breaks. HR differences were more notable during exercise, with LL‐BFR yielding lower HR. VT was consistently lower in LL‐BFR, matching the reductions in VE and VO_2_, while BR showed minimal differences. Participants reported higher RPE Leg but lower RPE Breathing under LL‐BFR compared to HL‐TRA throughout the session.

**TABLE 3:** Post‐hoc linear mixed models comparing mean differences in ventilation (VE) and secondary outcomes between high‐load resistance exercise (HL‐TRA) and low‐load blood flow restriction exercise (LL‐BFR).

	HL‐TRA	LL‐BFR	Mean difference	95% CI	% difference	*p*‐value adjusted
VE L/min						
Set 1	24.1	21.1	−3.02	−5.54 to −0.51	−8.4	0.030
Set 2	30.1	27.2	−2.93	−5.54 to −0.418	−9.7	0.030
Set 3	32.1	27.8	−4.25	−6.76 to −1.73	−13.2	0.004
Set 3 vs. Set 4	32.1	29.3	−2.80	−5.31 to −0.28	−8.7	0.040
Break 1	25.8	22.2	−3.58	−6.10 to −1.07	−13.9	0.014
Break 2	30.2	23.4	−6.79	−9.30 to −4.27	−22.5	<0.001
Post 1	27.3	24.0	−3.24	−5.76 to −0.73	−11.9	0.023
Post 2	16.2	14.4	−1.75	−4.29 to 0.80	−10.8	0.178
VO_2_ mL/min/kg						
Set 1	11.0	9.33	−1.68	−2.67 to 0.70	−15.3	0.001
Set 2	13.2	12.0	−1.21	−2.20 to −0.23	−9.2	0.026
Set 3	13.6	12.7	−0.91	−1.90 to 0.78	−6.7	0.071
Set 3 vs. Set 4	13.6	13.2	−0.44	−1.43 to 0.55	3.2	0.383
Break 1	13.3	10.1	−3.15	−4.14 to −2.16	−23.7	<0.001
Break 2	14.3	10.7	3.61	−4.60 to −2.26	−25.2	<0.001
Post 1	12.2	10.4	−1.73	−2.71 to −0.72	−14.2	<0.001
Post 2	6.30	5.65	−0.65	−1.65 to 0.35	−10.4	0.200
VO_2_ L/min						
Set 1	0.8	0.6	−0.12	−0.18 to −0.05	15.4	0.028
Set 2	0.9	0.8	−0.08	−0.15 to −0.02	10.3	0.020
Set 3	0.9	0.9	−0.06	−0.13 to −0.01	6.4	0.085
Set 3 vs. Set 4	0.9	0.9	−0.03	−0.04 to −0.01	3.2	0.396
Break 1	0.8	0.6	−0.12	−0.20 to −0.05	15.4	0.028
Break 2	0.9	0.8	−0.09	−0.15 to −0.02	10.3	0.020
Post 1	0.9	0.9	−0.06	−0.00831 to 0.127	6.4	0.085
Post 2	0.9	0.9	−0.03	−0.0975 to 0.0383	3.2	0.400
VCO_2_ L/min						
Set 1	0.7	0.6	−0.12	−0.20 to −0.05	17.4	0.002
Set 2	0.9	0.8	−0.13	−0.21 to −0.05	14.7	0.001
Set 3	0.9	0.8	−0.15	−0.22 to −0.07	15.5	<0.001
Set 3 vs. Set 4	0.9	0.8	−0.11	−0.19 to 0.03	11.7	0.005
Break 1	0.8	0.6	−0.22	−0.29 to −0.14	25.7	<0.001
Break 2	1.0	0.7	−0.32	−0.39 to −0.24	32.6	<0.001
Post 1	0.8	0.7	−0.13	−0.21 to −0.054	15.6	0.001
Post 2	0.4	0.3	−0.07	−0.15 to −0.01	16.3	0.072
RER						
Set 1	0.9	0.9	−0.03	−0.07 to 0.01	−3.2	0.171
Set 2	0.9	0.9	−0.02	−0.07 to 0.02	−2.5	0.263
Set 3	1.0	0.9	−0.06	−0.10 to −0.02	−0.63	0.004
Set 3 vs. Set 4	1.0	0.9	−0.03	−0.07 to 0.01	−6.6	0.228
Break 1	1.0	0.9	−0.12	−0.16 to −0.08	−11.7	<0.001
Break 2	1.1	0.9	−0.21	−0.25 to −0.17	−18.7	<0.001
Post 1	1.1	1.0	−0.07	−0.11 to −0.03	−6.3	0.002
Post 2	1.0	0.9	−0.08	−0.12 to −0.04	−8.3	<0.001
VE/VCO_2_						
Set 1	26.7	29.4	2.65	0.42 to 4.88	9.9	0.020
Set 2	27.0	29.6	2.60	0.37 to 4.83	9.6	0.022
Set 3	27.3	29.5	2.13	−0.10 to 4.36	7.8	0.098
Set 3 vs. Set 4	27.3	29.5	−2.18	−4.41 to 0.04	−8.0	0.055
Break 1	26.3	29.1	2.79	0.57 to 5.02	10.6	0.014
Break 2	27.1	29.6	2.45	0.22 to 4.68	9.0	0.031
Post 1	28.5	29.8	1.33	−0.897 to 3.56	4.7	0.241
Post 2	28.3	29.8	1.45	−0.78 to 3.67	5.1	0.203
VT L/min						
Set 1	1.1	0.9	−0.17	−0.30 to −0.05	15.6	0.016
Set 2	1.3	1.2	−0.10	−0.2 to 0.02	−9.45	0.129
Set 3	1.3	1.2	−0.10	−0.23 to 0.02	−7.87	0.129
Set 3 vs. Set 4	1.3	1.3	−0.06	−0.18 to 0.07	−4.33	0.380
Break 1	1.3	1.1	−0.21	−0.33 to −0.08	−16.0	0.006
Break 2	1.4	1.1	−0.29	−0.42 to −0.17	−20.8	<0.001
Post 1	1.3	1.1	−0.19	−0.31 to −0.06	−14.2	0.011
Post 2	0.9	0.7	−0.14	−0.27 to 0.01	−15.7	0.057
BR (breaths per min)						
Set 1	23.8	24.6	0.86	−0.96 to 2.69	3.5	0.539
Set 2	25.4	25.6	0.21	−1.61 to 2.04	0.8	0.911
Set 3	25.8	25.0	−0.78	−2.60 to 1.05	−3.1	0.403
Set 3 vs. Set 4	25.8	25.0	−0.78	−2.60 to 1.05	−3.1	0.404
Break 1	21.0	22.7	1.70	−0.13 to 3.52	7.8	0.451
Break 2	22.5	22.6	0.10	−1.72 to 1.93	0.5	0.911
Post 1	21.2	22.2	0.99	−0.84 to 2.81	4.4	0.539
Post 2	19.2	20.7	1.49	−0.35 to 3.34	7.2	0.451
SpO_2_						
Set 1	96.5	96.7	0.21	−0.23 to 0.63	0.2	0.608
Set 2	96.6	96.9	0.24	−0.19 to 0.67	0.3	0.608
Set 3	96.9	96.7	−0.13	−0.57 to 0.30	−0.1	0.541
Set 3 vs. Set 4	96.9	96.7	−0.19	−0.62 to 0.24	−0.2	0.380
Break 1	96.2	96.8	0.52	−0.089 to 0.95	0.5	0.146
Break 2	96.4	96.7	0.31	−0.12 to 0.74	0.3	0.608
Post 1	96.3	96.5	0.13	−0.31 to 0.56	0.1	0.650
Post 2	96.3	96.4	0.10	−0.34 to 0.54	0.1	0.653
HR (bpm)						
Set 1	109.0	99.6	−9.55	−15.0 to −4.10	−9.6	0.003
Set 2	111.0	101.0	−9.97	−15.4 to −4.52	−9.9	0.003
Set 3	111.0	103.0	−7.97	−13.4 to −2.52	−7.8	0.011
Set 3 vs. Set 4	111.0	105.0	−6.58	−12.0 to −1.14	−6.2	0.036
Break 1	92.7	94.4	1.70	−3.75 to 7.14	1.8	0.630
Break 2	97.0	96.3	−0.63	−6.08 to 4.81	−0.7	0.819
Post 1	95.6	91.4	−4.17	−9.62 to 1.28	−4.6	0.213
Post 2	81.3	79.6	−1.67	−7.18 to 3.84	−2.6	0.551
RPE Leg (0–10)						
Set 1	4.2	5.4	1.25	0.54 to 1.96	30.0	<0.001
Set 2	5.3	6.5	1.29	0.58 to 2.00	24.6	<0.001
Set 3	5.8	7.4	1.58	0.87 to 2.29	27.1	<0.001
Set 3 vs. Set 4	5.8	8.0	2.17	1.46 to 2.88	37.2	<0.001
Post 1	2.5	4.2	1.63	0.91 to 2.34	64.2	<0.001
Post 2	1.3	2.2	0.89	0.18 to 1.61	69	0.014
RPE breathing (0–10)						
Set 1	2.8	2.0	−0.88	−1.39 to −0.36	−31.1	<0.001
Set 2	3.8	2.4	−1.44	−1.95 to −0.92	−37.8	<0.001
Set 3	4.3	2.7	−1.6	−2.12 to −1.09	−37.3	<0.001
Set 3 vs. Set 4	4.3	3.0	−1.31	−1.82 to −0.80	−30.5	<0.001
Post 1	1.3	1.1	−0.25	−0.26 to 0.76	−19.1	0.337
Post 2	0.8	0.5	−0.23	−0.26 to 0.76	−29.9	0.380

*Note*: Data are presented as means with corresponding 95% confidence intervals, mean differences with corresponding 95% confidence intervals, and percent differences with corresponding 95% confidence intervals. Positive coefficients indicate that high‐load resistance exercise resulted in larger measurements compared to low‐load blood flow restriction exercise. *p*‐values were adjusted for multiple testing using the Benjamini‐Hochberg correction.

Abbreviations: BR, breathing rate; RPE breathing, ratings of perceived breathing on a scale of 0–10 (0 no shortness of breath 10 maximum shortness of breath); RPE Leg, rating of perceived leg exertion on a scale of 0–10 (0 no fatigue; 10 maximum fatigue); SpO_2_, peripheral oxygen saturation; VCO_2_, carbon dioxide output; VE, ventilation; VO_2_, oxygen consumption; VT, tidal volume.

## 4 DISCUSSION

This randomized crossover study compared the acute respiratory and perceptual responses to a single session of LL‐BFR and HL‐TRA in healthy adults. In line with our hypothesis, LL‐BFR elicited significantly lower respiratory demands than HL‐TRA, as reflected by reduced V̇E, V̇O_2_, V̇CO_2_, V̇T, and heart rate. These findings indicate that, despite its potent muscular stimulus, LL‐BFR was associated with lower acute respiratory demands compared to HL‐TRA under the conditions of this study. Notably, the observed difference in VE (−3.32 L/min) is unlikely to be clinically relevant in healthy individuals, and whether such differences translate into meaningful benefits in clinical populations remains unknown. While previous research has primarily focused on the chronic muscular adaptations associated with LL‐BFR, comparatively less attention has been paid to its acute respiratory demands during resistance exercise (Gronfeldt et al., 2020; Hughes et al., 2017; Lixandrao et al., 2018).

Existing studies on BFR endurance exercise suggest reduced respiratory strain compared to traditional endurance protocols; no prior work has assessed these responses during BFR resistance exercise (Kilgas et al., 2022; Kuhn et al., 2024). By characterizing ventilatory and metabolic responses during a single session of established BFR resistance exercise, following current guidelines, the present study extends the literature and provides insight into the acute respiratory demands of LL‐BFR (Patterson, Hughes, Warmington, et al., 2019). The investigation in healthy individuals was a deliberate methodological choice to establish baseline physiological responses under controlled conditions. While these results are applicable to healthy adults under the conditions of this study, they do not allow direct conclusions regarding clinical populations.

Our findings were consistent across both ventilatory and perceptual measures and were supported by a sensitivity analysis assessing mean differences during the final 20 s of each exercise phase (Tables S1 and S2).

To further contextualize ventilatory responses, we analyzed ventilatory equivalents (V̇E/V̇CO_2_) and respiratory exchange ratio (RER). Although V̇E/V̇CO_2_ was higher during LL‐BFR compared to HL‐TRA, this finding should be interpreted with caution. During LL‐BFR, venous outflow from the exercising limb is intentionally restricted, which can delay the transport of metabolically produced CO_2_ to the lungs. As a result, pulmonary V̇CO_2_ measurements obtained during occlusion may underestimate true metabolic CO_2_ production. This underestimation may artificially elevate V̇E/V̇CO_2_ and does not necessarily reflect reduced ventilatory efficiency or increased ventilatory load. This interpretation is supported by the lower RER observed during LL‐BFR, indicating reduced apparent CO_2_ output despite lower overall metabolic demand. Together, these findings suggest that the relationship between ventilation and CO_2_ output is altered during LL‐BFR due to the unique physiological effects of venous occlusion. Therefore, V̇E/V̇CO_2_ in this context should be interpreted as reflecting altered CO_2_ kinetics rather than a direct marker of ventilatory efficiency. Consequently, direct comparisons of ventilatory efficiency between LL‐BFR and HL‐TRA using V̇E/V̇CO_2_ are limited and should be interpreted cautiously.

Differences between LL‐BFR and HL‐TRA were more pronounced during the break phases, particularly during Break 2, compared to the set phases. This pattern was observed across several respiratory variables, including V̇E, V̇O_2_, V̇CO_2_, and V̇T, despite shorter rest intervals in the LL‐BFR condition. Notably, the higher VCO_2_ observed during the break periods in HL‐TRA compared to LL‐BFR may further support the concept of altered CO_2_ transit kinetics during venous occlusion. One potential explanation relates to sustained venous restriction during LL‐BFR, which may temporarily impair metabolite and CO_2_ washout from the exercising limb during recovery periods, whereas HL‐TRA may allow more immediate clearance between sets. However, as these mechanisms were not directly assessed, this interpretation remains speculative. An additional explanation may relate to breathing patterns, including the Valsalva maneuver, which is known to increase with exercise intensity (Hackett & Chow, 2013; MacDougall et al., 1992). This maneuver increases intrathoracic pressure and may transiently affect lung volumes and gas exchange. (Levitzky, 2013) As a result, respiratory responses during recovery phases may reflect both metabolic and mechanical factors. However, as breathing patterns were not directly measured, this explanation should also be considered speculative.

Heart rates during the set phases were approximately 6 to 10 bpm higher in the HL‐TRA condition compared to LL‐BFR. This contrasts with previous studies reporting higher heart rate responses during BFR exercise under matched workload conditions (Lemos et al., 2022; Ozaki et al., 2010; Poton & Polito, 2016). These differences may relate to the substantially higher absolute load used in the HL‐TRA condition in the present study (approximately 390 kg), reflecting a pragmatic training approach. Accordingly, differences in exercise loading should be considered when interpreting these findings.

RPE‐Leg was higher during LL‐BFR compared to HL‐TRA. This differs from previous findings under fixed repetition schemes but is consistent with studies showing greater perceived discomfort during LL‐BFR when exercises are performed close to failure (de Queiros et al., 2022). In the present study, the LL‐BFR protocol was designed to approach, but not reach, muscular failure, which may explain the higher perceived exertion.

RPE‐Breathing was lower during LL‐BFR compared to HL‐TRA. In healthy individuals, ventilatory demands are unlikely to limit exercise, and perceptions of breathlessness are more closely related to neural respiratory drive than to absolute ventilatory load (Molgat‐Seon et al., 2019; Yates, 2019). Thus, the observed difference likely reflects perceptual rather than physiological factors. These observations may warrant further investigation in clinical populations in whom dyspnea limits exercise. Previous work has reported reduced RPE‐Breathing with LL‐BFR in patients with COPD, and potential applications in cardiovascular populations have been suggested (Cahalin et al., 2022; Kohlbrenner et al., 2023). However, such implications remain speculative and require direct investigation.

Although workloads were not matched between conditions, both protocols followed established guidelines, supporting their relevance to real‐world practice (Liguori et al., 2021; Patterson, Hughes, Warmington, et al., 2019). The decision not to match absolute loads reflects typical training approaches, although it limits direct mechanistic comparisons between conditions.

Several limitations should be acknowledged. First, inherent differences in protocol design, including the absence of a low‐load non‐BFR condition as well as differences in external load and repetition structure, limit the ability to fully isolate the independent effects of mechanical load and vascular occlusion and restrict direct mechanistic comparisons. Second, only acute responses were assessed, and it remains unclear whether these differences translate to chronic adaptations. Third, limb occlusion pressure was determined in the supine position while exercise was performed seated, which may influence applied pressure. Fourth, one‐repetition maximum (1RM) was estimated rather than directly measured, in accordance with ACSM guidelines (Liguori et al., 2021). Fifth, residual fatigue from 1RM testing may have influenced responses despite the crossover design. Although blood flow restriction pressure was individualized using the Delfi Personalized Tourniquet System, direct verification of arterial occlusion during exercise using imaging techniques such as Doppler ultrasound was not performed.

## 5 CONCLUSION

In conclusion, this study compared the acute respiratory responses to resistance exercise between LL‐BFR and HL‐TRA in healthy individuals. Under the conditions of this study, LL‐BFR was associated with lower values in key respiratory parameters compared to HL‐TRA, alongside lower perceived breathing effort but higher perceived leg exertion. These findings suggest that LL‐BFR and HL‐TRA elicit distinct physiological and perceptual response profiles. However, due to protocol differences and the absence of a low‐load non‐BFR condition, the findings should be interpreted as comparisons between two exercise paradigms rather than isolated effects of BFR. Future studies are warranted to further investigate these responses in clinical populations.

## AUTHOR CONTRIBUTIONS

**Manuel Kuhn:** Conceptualization; data curation; formal analysis; funding acquisition; investigation; methodology; project administration; visualization. **Dario Kohlbrenner:** Conceptualization; funding acquisition; methodology; validation. **Adrian Kläy:** Data curation; formal analysis; investigation. **Malcolm Kohler:** Supervision; validation. **Alea Gheza:** Investigation; project administration. **Laura C. Mayer:** Methodology; validation. **Thomas Radtke:** Methodology; validation. **Sarah R. Haile:** Data curation; formal analysis; validation. **Diego M. Baur:** Data curation; formal analysis; methodology; validation. **Noriane A. Sievi:** Validation; methodology. **Christian F. Clarenbach:** Funding acquisition; project administration; supervision.

## FUNDING INFORMATION

Lung Zurich (2021‐03); Recipient's: Christian Clarenbach and Dario Kohlbrenner.

Heubergstiftung (09‐2022); Recipient's: Manuel Kuhn.

## CONFLICT OF INTEREST STATEMENT

N. A. Sievi, D. Kohlbrenner., A. Kläy, M. Luchinger, A. Gheza, T. Radtke, S. R. Haile, L. Mayer, Diego Baur and M. Kuhn have no conflicts of interests. M. Kohler received advisory fees from Roche Diagnostics, GSK and Novartis. C. F. Clarenbach received advisory fees from Roche, Novartis, Boehringer, GSK, Astra Zeneca, Sanofi, Vifor, OM Pharma, CSL Behring, Grifols, Daiichi Sankyo and MSD within the last 36 months.

## Supporting information


**Table S1.** Linear mixed models on difference in mean VE and secondary outcomes between traditional and BFR strength exercise over Set1, Set2, Set3, Break1, and Break 2 (means final 20 s of each phase).
**Table S2.** Post‐hoc linear mixed models on difference in mean VE and secondary outcomes between traditional and BFR endurance exercise in the individual exercise phases (means final 20 s of each phase).

## Data Availability

The data that support the findings of this study are available from the corresponding author, Manuel Kuhn, upon request.
