# Supplementary material for: The acute respiratory response to blood‐flow restriction resistance exercise in healthy adults: A randomized crossover trial
Source: Physiol Rep. 2026 Jun 12;14(11):e70968. doi: 10.14814/phy2.70968 (PMC13261080; doi:10.14814/phy2.70968)
Supplement: Supplementary file 1 — Table S1. Linear mixed models on difference in mean VE and secondary outcomes between traditional and BFR strength exercise over Set1, Set2, Set3, Break1, and Break 2 (means final 20 s of each phase). Table S2. Post‐hoc linear mixed models on difference in mean VE and secondary outcomes between traditional and BFR endurance exercise in the individual exercise phases (means final 20 s of each phase). [file PHY2-14-e70968-s001.docx]

| **Supplement Table 1\| Linear mixed models on difference in mean VE and secondary outcomes between traditional and BFR strength exercise over Set1, Set2, Set3, Break1 and Break 2 (Means final 20sec of each phase)** | | | | | | | | | | | |
| --- | --- | --- | --- | --- | --- | --- | --- | --- | --- | --- | --- |
|  | **TRA** | **BFR** | **Mean Difference** | | **95% CI** | | **% Difference** | | **P Value** | | |
| VE L/min | 27.4 (1.23) | 25.5 (1.20) | | -1.94 | | -3.15 – -0.72 | | -7.08% | | 0.002 |  |
| VO2 L/min | 0.83 (0.04) | 0.78 (0.04) | | -0.05 | | -0.08 – -0.01 | | -6.02% | | 0.013 |  |
| VO2 kg/L/min | 12.1 (0.48) | 11.4 (0.46) | | -0.66 | | -1.20 – -0.12 | | -5.45% | | 0.018 |  |
| VCO2 L/min | 0.816 (0.04) | 0.711 (0.04) | | -0.10 | | -0.14 – -0.07 | | -12.25% | | **<0.001** |  |
| VT L | 1.19 (0.08) | 1.13 (0.08) | | -0.06 | | -0.12 – 0.00 | | -5.04% | | 0.036 |  |
| BR (breaths per min) | 24.3 (1.00) | 24.6 (0.98) | | 0.33 | | -0.59 – 1.25 | | 1.35% | | 0.484 |  |
| RER | 0.988 (0.02) | 0.906 (0.01) | | -0.08 | | -0.10 – -0.06 | | -8.09% | | **<0.001** |  |
| Heart rate (bpm) | 102.3 (2.01) | 99.9 (1.86) | | -2.35 | | -5.80 – 1.10 | | -2.29% | | 0.181 |  |
| SpO2 (%) | 96.5 (0.19) | 96.7 (0.19) | | 0.15 | | -0.04 – 0.35 | | 0.16% | | 0.122 |  |
| RPE leg (0-10) | 5.08 (0.33) | 6.84 (0.32) | | 1.76 | | 1.32 – 2.20 | | 34.65% | | **<0.001** |  |
| RPE breathing (0-10) | 3.64 (0.27) | 2.50 (0.27) | | -1.15 | | -1.45 – -0.84 | | 31.59% | | **<0.001** |  |
| NOTE. Data are presented as means with corresponding 95% confidence intervals, mean differences with corresponding 95% confidence intervals, and percent differences with corresponding 95% confidence intervals. Positive coefficients indicate that high-load resistance exercise resulted in larger measurements compared to low-load blood flow restriction exercise. P-values were adjusted for multiple testing using the Benjamini-Hochberg correction.  Abbreviations: VE: Ventilation; VO2: Oxygen consumption; VCO2: Carbon dioxide output; VT: Tidal volume; BR: Breathing rate; RER: Respiratory exchange ratio SpO2: Peripheral oxygen saturation; RPE leg: Rating of perceived leg exertion on a scale of 0 to 10 (0 no fatigue; 10 maximum fatigue); RPE dyspnea: Ratings of perceived dyspnea on a scale of 0 to 10 (0 no shortness of breath 10 maximum shortness of breath) | | | | | | | | | | | |

| **Supplement Table 2 \| Post-hoc linear mixed models on difference in mean VE and secondary outcomes between traditional and BFR endurance exercise in the individual exercise phases (Means final 20sec of each phase)** | | | | | | |
| --- | --- | --- | --- | --- | --- | --- |
|  | **TRA** | **BFR** | **Mean Difference** | **95% CI** | **% Difference** | **P-Value corrected** |
| **Set1** | | | | | | |
| VE L/min | 24.9 (1.39) | 23.5 (1.38) | -1.36 | -3.97 - 1.24 | -5.46% | 0.304 |
| VO_2_ L/min | 0.81 (0.04) | 0.73 (0.04) | - 0.01 | -0.005 – -0.152 | -1.23% | **0.037** |
| VO_2_ mL/kg/min | 11.9 (0.54) | 10.8 (0.53) | 1.07 | -0.03 – 2.18 | 8.99% | 0.056 |
| VCO_2_ L/min | 0.73 (0.04) | 0.65 (0.04) | -0.08 | -0.01 – -0.16 | -10.96% | 0.031 |
| VT L | 1.14 (0.08) | 1.09 (0.08) | 0.05 | -0.08 – 0.18 | 4.39% | 0.486 |
| BR (breaths per min) | 23.6 (1.17) | 24.6 (1.16) | -1.04 | -3.28 – 1.21 | -4.41% | 0.365 |
| RER | 0.9 (0.02) | 0.885 (0.02) | 0.02 | -0.03 – 0.06 | 2.22% | 0.480 |
| Heart rate (bpm) | 114 (2.57) | 105 (2.53) | 8.90 | 3.01 – 14.8 | 7.81% | 0.003 |
| SpO2 (%) | 96.5 (0.24) | 96.9 (0.24) | -0.43 | -0.91 – 0.05 | -0.45% | 0.078 |
| RPE leg (0-10) | 4.17 (0.32) | 5.42 (0.32) | -1.25 | -1.96 – -0.54 | -29.98% | **<0.001** |
| RPE breathing (0-10) | 2.83 (0.25) | 1.96 (0.25) | -0.88 | -0.36 – -1.39 | -31.09% | **<0.001** |
| **Set2** | | | | | | |
| VE L/min | 30.0 (1.39) | 27.3 (1.38) | -2.65 | -5.25 - -0.0437 | -8.83% | 0.044 |
| VO2 L/min | 0.96 (0.04) | 0.82 (0.04) | -0.13 | -0.06 – -0.21 | -13.54% | **<0.001** |
| VO2 mL/kg/min | 14.0 (0.54) | 12.1 (0.53) | 1.93 | 0.83 – 3.03 | 13.79% | **<0.001** |
| VCO2 L/min | 0.89 (0.04) | 0.76 (0.04) | -0.14 | -0.06 –0-0.21 | -15.73% | **<0.001** |
| VT L | 1.25 (0.08) | 1.19 (0.08) | 0.06 | -0.07 – 0.19 | 4.8% | 0.362 |
| BR (breaths per min) | 25.5 (1.17) | 25.3 (1.16) | 0.197 | -2.05 – 2.44 | 0.77% | 0.863 |
| RER | 0.94 (0.02) | 0.92 (0.02) | 0.01 | -0.03 – 0.05 | 1.06% | 0.545 |
| Heart rate (bpm) | 116 (2.57) | 103 (2.57) | 12.9 | 7.01 – 18.8 | 11.12% | **<0.001** |
| SpO2 (%) | 96.5 (0.24) | 96.8 (0.24) | -0.28 | -0.76 – 0.19 | -0.29% | 0.246 |
| RPE leg (0-10) | 5.25 (0.32) | 6.54 (0.32) | -1.29 | -2.00 – -0.58 | -24.57% | **<0.001** |
| RPE breathing (0-10) | 3.81 (0.25) | 2.38 (0.25) | -1.44 | -0.92 – -1.95 | -37.79% | **<0.001** |
| **Set3** | | | | | | |
| VE L/min | 31.8 (1.39) | 28.2 (1.38) | -3.62 | -6.22 - -1.01 | -11.38% | 0.0198 |
| VO2 L/min | 0.94 (0.04) | 0.87 (0.04) | - 0.12 | -0.05 – -0.19 | -12.77% | **<0.001** |
| VO2 mL/kg/min | 14.5 (0.54) | 12.7 (0.53) | 1.78 | 0.68 – 2.89 | 12.28% | 0.002 |
| VCO2 L/min | 0.95(0.04) | 0.79 (0.04) | -0.16 | -0.08 – -0.23 | -16.84% | **<0.001** |
| VT L | 1.31 (0.08) | 1.20 (0.08) | 0.11 | -0.02 – 0.25 | 8.39% | 0.103 |
| BR (breaths per min) | 25.6 (1.17) | 25.4 (1.16) | 0.208 | -2.04 – 2.45 | 0.81% | 0.856 |
| RER | 0.96 (0.02) | 0.91 (0.02) | 0.05 | 0.01 – 0.09 | 5.21% | 0.017 |
| Heart rate (bpm) | 116 (2.57) | 106 (2.53) | 10.4 | 4.53 – 16.3 | 8.97% | **<0.001** |
| SpO2 (%) | 96.6 (0.24) | 96.6 (0.24) | -0.02 | -0.5 – 0.46 | -0.02% | 0.926 |
| RPE leg (0-10) | 5.83 (0.32) | 7.42 (0.32) | -1.58 | -2.29 – -0.87 | -27.10% | **<0.001** |
| RPE breathing (0-10) | 4.29 (0.25) | 2.69 (0.25) | -1.6 | -1.09 – -2.12 | -37.29% | **<0.001** |
| **Break1** | | | | | | |
| VE L/min | 23.9 (1.2) | 22.1 (1.4) | -1.71 | -4.31 - 0.895 | -7.15% | 0.237 |
| VO_2_ L/min | 0.69 (0.04) | 0.68 (0.04) | -0.01 | -0.06 – 0.09 | -1.45% | 0.749 |
| VO_2_ mL/kg/min | 10.1 (0.54) | 9.88 (0.53) | 0.18 | -0.92 – 1.29 | 1.78% | 0.742 |
| VCO_2_ L/min | 0.72 (0.04) | 0.62 (0.04) | -0.09 | -0.02 –-0.18 | -12.5% | 0.011 |
| VT L | 1.08 (0.08) | 0.99 (0.08) | 0.09 | -0.04 – 0.23 | 8.33% | 0.172 |
| BR (breaths per min) | 23 (1.17) | 24 (1.16) | -1.06 | -3.30 – 1.19 | -4.61% | 0.356 |
| RER | 1.04 (0.02) | 0.91 (0.02) | 0.123 | 0.08 – 0.16 | 11.82% | **<0.001** |
| Heart rate (bpm) | 82.4 (2.57) | 90.3 (2.53) | -7.85 | -13.7 – -1.96 | -9.53% | **<0.001** |
| SpO2 (%) | 96.5 (0.24) | 96.6 (0.24) | -0.17 | -0.65 – 0.31 | -0.18% | 0.493 |
| **Break2** | | | | | | |
| VE L/min | 27.3 (1.39) | 23.2 (1.38) | -4.07 | -6.67– -1.47 | -14.91% | 0.0135 |
| VO2 L/min | 0.72 (0.04) | 0.71 (0.04) | -0.007 | -0.066 – 0.081 | -0.97% | 0.840 |
| VO2 mL/kg/min | 10.5 (0.54) | 10.3 (0.53) | 0.134 | -0.97 – 1.24 | 1.28% | 0.812 |
| VCO2 L/min | 0.80 (0.04) | 0.64 (0.04) | -0.16 | -0.08 – -0.24 | -20.00% | **<0.001** |
| VT L | 1.18 (0.08) | 1.04 (0.08) | 0.14 | 0.01 – 0.28 | 11.86% | 0.039 |
| BR (breaths per min) | 24 (1.17) | 23.9 (1.16) | 0.09 | -2.15 – 2.34 | 0.38% | 0.935 |
| RER | 1.10 (0.02) | 0.90 (0.02) | 0.20 | 0.16 – 0.25 | 18.18% | **<0.001** |
| Heart rate (bpm) | 86 (2.57) | 93 (2.53) | -6.76 | -12.7 – -0.87 | -7.86% | 0.025 |
| SpO2 (%) | 96.6 (0.24) | 96.6 (0.24) | -0.03 | -0.51 – 0.45 | -0.03% | 0.907 |
| **Traditional Set3 vs. BFR Set4** | | | | | | |
| VE L/min | 31.8 (1.39) | 28.8 (1.38) | -3.00 | -5.60– -0.40 | -9.43% | 0.0482 |
| VO2 L/min | 0.99 (0.04) | 0.98 (0.04) | -0.09 | -0.01 – -0.16 | -9.09% | 0.019 |
| VO2 mL/kg/min | 14.5 (0.54) | 13.1 (0.53) | 1.33 | 0.22 – 2.43 | 9.17% | 0.018 |
| VCO2 L/min | 0.95 (0.04) | 0.82 (0.04) | -0.13 | -0.05 – -0.21 | -13.68% | **<0.001** |
| VT L | 1.31 (0.08) | 1.28 (0.08) | 0.03 | -0.10 – 0.17 | 2.29% | 0.653 |
| BR (breaths per min) | 25.6 (1.17) | 24.8 (1.16) | 0.81 | -1.43 – 3.06 | 3.16% | 0.476 |
| RER | 0.96 (0.02) | 0.91 (0.02) | 0.06 | 0.01 – 0.09 | 6.25% | 0.009 |
| Heart rate (bpm) | 116 (2.57) | 106 (2.53) | 9.64 | 3.75 – 15.5 | 8.31% | 0.0014 |
| SpO2 (%) | 96.6 (0.24) | 96.8 (0.24) | -0.21 | -0.69 – 0.27 | -0.22% | 0.399 |
| RPE leg (0-10) | 5.83 (0.32) | 8.00 (0.32) | -2.17 | -2.88 – -1.46 | -37.22% | **<0.001** |
| RPE breathing (0-10) | 4.29 (0.25) | 2.98 (0.25) | -1.31 | -0.80 – -1.82 | -30.54% | **<0.001** |
| **Post1** | | | | | | |
| VE L/min | 20.2 (1.39) | 18.7 (1.38) | -1.55 | -4.31- -0.9 | -7.67% | 0.278 |
| VO2 L/min | 0.51 (0.04) | 0.48 (0.04) | -0.03 | -0.05 –0-0.10 | -5.88% | 0.454 |
| VO2 mL/kg/min | 7.47 (0.54) | 6.94 (0.53) | 0.53 | -0.57 – 1.64 | 7.09% | 0.342 |
| VCO2 L/min | 0.55 (0.04) | 0.50 (0.04) | -0.05 | 0.02 – -0.13 | -9.09% | 0.143 |
| VT L | 1.04 (0.08) | 0.86 (0.08) | 0.18 | 0.05 – 0.31 | 17.31% | 0.009 |
| BR (breaths per min) | 20.4 (1.17) | 22.3 (1.16) | -1.97 | -4.21 – 0.28 | -9.66% | 0.086 |
| RER | 1.08 (0.02) | 1.02 (0.02) | 0.05 | 0.012 – 0.096 | 4.62% | 0.0012 |
| Heart rate (bpm) | 83.6 (2.57) | 84.4 (2.53) | -0.75 | -6.64 – 5.14 | -0.89% | 0.802 |
| SpO2 (%) | 96.5 (0.24) | 96.6 (0.24) | -0.12 | -0.60 – 0.36 | -0.12% | 0.622 |
| RPE leg (0-10) | 2.54 (0.32) | 4.17 (0.32) | -1.63 | -2.34 – -0.91 | -64.17% | **<0.001** |
| RPE breathing (0-10) | 1.31 (0.25) | 1.06 (0.25) | -0.25 | 0.26 – -0.76 | -19.08% | 0.337 |
| **Post2** | | | | | | |
| VE L/min | 15.2 (1.39) | 13.5 (1.38) | -1.62 | -4.22- -0.981 | -10.66% | 0.278 |
| VO2 L/min | 0.41 (0.04) | 0.37 (0.04) | -0.04 | -0.04 – 0.11 | -9.76% | 0.034 |
| VO2 mL/kg/min | 5.98 (0.54) | 5.40 (0.53) | 0.57 | -0.53 – 1.68 | 9.53% | 0.309 |
| VCO2 L/min | 0.39 (0.04) | 0.34 (0.04) | -0.06 | 0.015 – -0.14 | -15.38% | 0.120 |
| VT L | 0.84 (0.08) | 0.65 (0.08) | 0.19 | 0.05 – 0.32 | 22.61% | 0.006 |
| BR (breaths per min) | 18.7 (1.17) | 23.9 (1.16) | -2.57 | -2.15 – 2.34 | -13.74% | 0.025 |
| RER | 0.97 (0.02) | 0.89 (0.02) | 0.08 | 0.04 – 0.12 | 8.25% | **<0.001** |
| Heart rate (bpm) | 81.9 (2.57) | 77.5 (2.53) | 4.73 | -1.52 – 10.3 | 5.77% | 0.146 |
| SpO2 (%) | 96.2 (0.24) | 96.2 (0.24) | -0.07 | -0.55 – 0.41 | -0.07% | 0.768 |
| RPE leg (0-10) | 1.29 (0.32) | 2.19 (0.32) | -0.89 | -1.61 – -0.18 | -68.99% | 0.014 |
| RPE breathing (0-10) | 0.77 (0.25) | 0.54 (0.25) | -0.23 | 0.26 – -0.76 | -29.87% | 0.38 |
| *NOTE. Data are presented as means with corresponding 95% confidence intervals, mean differences with corresponding 95% confidence intervals, and percent differences with corresponding 95% confidence intervals. Positive coefficients indicate that high-load resistance exercise resulted in larger measurements compared to low-load blood flow restriction exercise. P-values were adjusted for multiple testing using the Benjamini-Hochberg correction.*  *. Abbreviations: VE: Ventilation; VO_2_: Oxygen consumption; VCO_2_: Carbon dioxide output; VT: Tidal volume; BR: Breathing rate; RER: Respiratory exchange ratio SpO_2_: Peripheral oxygen saturation; RPE leg: Rating of perceived leg exertion on a scale of 0 to 10 (0 no fatigue; 10 maximum fatigue); RPE dyspnea: Ratings of perceived dyspnea on a scale of 0 to 10 (0 no shortness of breath 10 maximum shortness of breath)* | | | | | | |
